# Response of Fish Communities to Various Environmental Variables across Multiple Spatial Scales

**DOI:** 10.3390/ijerph9103629

**Published:** 2012-10-15

**Authors:** Yong-Su Kwon, Fengqing Li, Namil Chung, Mi-Jung Bae, Soon-Jin Hwang, Myeong-Seop Byoen, Sang-Jung Park, Young-Seuk Park

**Affiliations:** 1 Department of Biology, Kyung Hee University, Seoul 130-701, Korea; Email: davy3021@daum.net (Y.-S.K.); qflee3@gmail.com (F.L.); namilchung@gmail.com (N.C.); mjbae@khu.ac.kr (M.-J.B.); 2 Department of Environmental Science, Konkuk University, Seoul 143-701, Korea; Email: sjhwang@konkuk.ac.kr; 3 National Institute of Environmental Research, Incheon 407-708, Korea; Email: zacco@korea.kr (M.-S.B.); parkjoe@korea.kr (S.-J.P.)

**Keywords:** fish assemblage, community patterns, prediction, multiple spatial scales, self-organizing map, random forest, theoretical path model, indicator species

## Abstract

A better understanding of the relative importance of different spatial scale determinants on fish communities will eventually increase the accuracy and precision of their bioassessments. Many studies have described the influence of environmental variables on fish communities on multiple spatial scales. However, there is very limited information available on this topic for the East Asian monsoon region, including Korea. In this study, we evaluated the relationship between fish communities and environmental variables at multiple spatial scales using self-organizing map (SOM), random forest, and theoretical path models. The SOM explored differences among fish communities, reflecting environmental gradients, such as a longitudinal gradient from upstream to downstream, and differences in land cover types and water quality. The random forest model for predicting fish community patterns that used all 14 environmental variables was more powerful than a model using any single variable or other combination of environmental variables, and the random forest model was effective at predicting the occurrence of species and evaluating the contribution of environmental variables to that prediction. The theoretical path model described the responses of different species to their environment at multiple spatial scales, showing the importance of altitude, forest, and water quality factors to fish assemblages.

## 1. Introduction

The distribution and abundance of aquatic communities are governed by various environmental factors at different spatial scales [1,2,3,4]. Among aquatic organisms, fish are relatively easy to identify, and are an important component of aquatic ecosystems through their regulatory effects on a variety of ecosystem-level properties and functions via their consumption of lower trophic levels [5,6,7]. They are commonly recognized as sensitive keystone communities that can indicate habitat change, environmental degradation, and overall ecosystem health [8,9,10].

Diverse studies have explored the relationships between biotic and abiotic factors, including geological factors [11], land cover and land use types [12,13], hydrological factors [14], stream habitat characteristics [15], stream order [16,17,18,19], and water quality [20]. These environmental factors are considered in a hierarchical structure ranging from large scale to small scale. Large-scale factors (*i.e.*, landscape features) affect small-scale factors (*i.e.*, microhabitat conditions and water quality, which have important influences on the distribution and abundance of organisms). Therefore, environmental conditions can be viewed as constituting filters through which species in the regional species pool must pass to potentially be present at a given locale [21,22]. The multi-scale habitat filter primarily specifies a set of four habitat levels (watershed, reach, channel unit, and microhabitat). However, slightly different numbers of habitat levels and diversity of elements within levels have been reported [23,24]. Therefore, various studies have been carried out to predict fish distribution or to identify the important environmental factors affecting the distribution patterns of fish [25,26,27]. Predicting fish assemblages is relevant to the evaluation of environmental quality and is an important framework for ecological studies on species interactions [28]. Species composition models may support environmental management by simulating different environmental scenarios and pointing out the most critical factors that need to be changed or regulated [28].

Understanding the effects of environmental variables on the distribution of biodiversity is fundamental for developing biological monitoring tools. A better understanding of the relative importance of determinants of fish communities at different spatial scales will eventually increase the accuracy and precision of bioassessments [1]. Many studies have examined the influence of environmental variables on fish communities from Europe [25,29], North America [30,31], and Oceania [32,33]. However, very little information is available on the East Asian monsoon region, particularly Korea [34,35], despite this region’s long history and environmental features that have contributed to a rich biodiversity [1]. The Asian monsoon region has more than half of the World’s population and comprises a major portion of the largest ocean and the largest continent, including the highest mountains in the World [36].

In this study, we evaluated the relationship between fish communities and environmental variables at 691 sampling sites throughout South Korea. Our goals were as follows: (1) to characterize the distributional patterns of fish communities on the national scale, (2) to identify the most important environmental factors influencing the distribution and abundance of fish species for different environmental categories across multiple spatial scales, and (3) to clarify the relative influence of regional and local variables on fish community composition in Korean rivers.

## 2. Materials and Methods

### 2.1. Ecological Data

**Figure 1:**
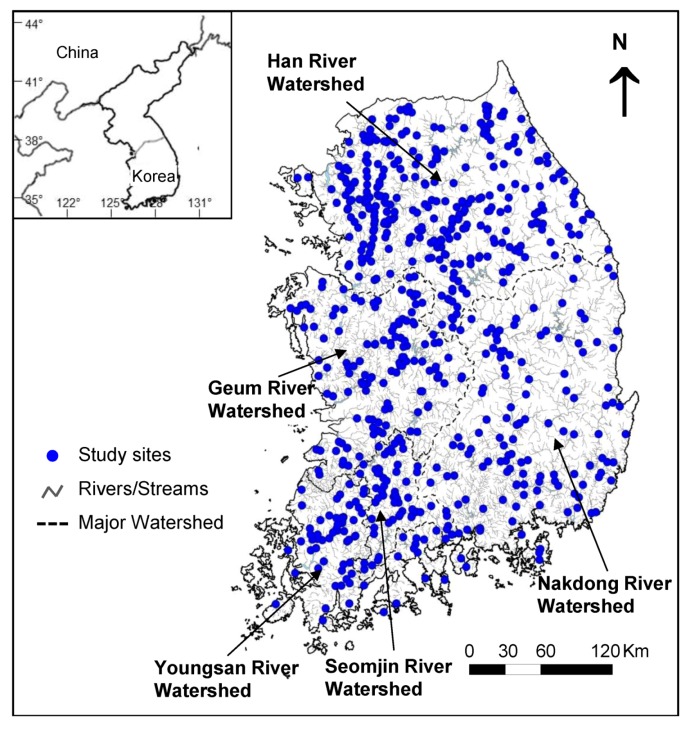
Locations of the sampling sites in five major watersheds in South Korea.

Fish community data were obtained from the National Aquatic Ecological Monitoring Program operated by the Ministry of Environment and the National Institute of Environmental Research, Korea. Fish were sampled at 720 sites from 388 rivers at the national scale (Figure 1) in April and May 2009, according to the standardized sampling protocol of the programme [37]. Among them, fish were collected at 691 sites. Five major rivers (Han, Nakdong, Geum, Yeongsan, and Seomjin Rivers) and their tributaries and small streams form the entire stream system in the country.

**Table 1:** Mean and standard deviation of 14 environmental variables in 691 sampling sites.

Category	Variable	Mean (±SD)
Geo-hydrological factors	Altitude (m)	108.7 (±127.7)
	Slope (%)	13.4 (±18)
	Stream Order	5.3 (±1.5)
	Distance from Source (DFS; km)	51.5 (±80.4)
Land cover types	Urban (%)	17 (±23.1)
	Forest (%)	30.1 (±30.7)
	Paddy field (%)	27.6 (±27.3)
	Dry field (%)	12.2 (±14.5)
Physicochemical factors	pH	8.1 (±0.8)
	Conductivity (EC; *μ*S/cm)	288.2 (±232.5)
	BOD (mg/L)	2.9 (±2.6)
	TN (mg/L)	2.9 (±2.4)
	TP (mg/L)	0.1 (±0.2)
	Chlorophyll-*a* (*μ*g/L)	5.3 (±10.8)

Fish were collected from all types of habitats in each site, including riffle, run, and pool areas, based on the catch per unit effort, with two types of sampling equipment: casting net (5-mm mesh size) and kick net (4-mm mesh size). Stream segments of approximately 200 m were sampled at each site for 50 min. Most of the captured fish were identified to the species level in the field. Among the collected specimens, some species requiring detailed identification and observation were fixed with 10% formalin solution and transported to the laboratory. Details of the sampling protocol for fish are described in MOE/NEIR [37].

Fourteen environmental variables were measured at each site. Environmental variables were categorised into three groups, geo-hydrological factors, land cover types, and physicochemical factors to indicate different fish assemblage characteristics (Table 1). Slope and stream order were obtained from the Water Management Information System (WAMIS, http://www.wamis.go.kr) of the Ministry of Land, Transport and Maritime Affairs, Korea. Altitude was measured from a Digital Elevation Map (DEM), and distance from the source (DFS) was calculated as the distance from the source of the stream to each site. The above variables were extracted from a digital map using ArcGIS 9.3 (www.esri.com). Land cover types (urban area, forest area, paddy field, and dry field) were obtained from the Ministry of Environment, Korea. The proportion of each type of land cover was extracted from a 1,000 × 100 m (length × width) area that included each sampling site on a digital map using ArcGIS 9.3 (www.esri.com). Dissolved oxygen (DO), conductivity, and pH were measured in the field using YSI 85 meters (YSI Inc., Yellow Springs, OH, USA) for DO and conductivity, and an Orion 3-Star-Plus pH meter (Thermo Fisher Scientific Inc., Waltham, MA, USA), for pH. Other variables, such as biological oxygen demand (BOD), total nitrogen (TN), and total phosphorus (TP), were analyzed in the laboratory by using the techniques by Eaton *et al.* [38]. Details of the sample collection protocol for fish and that for laboratory analysis are described in MOE/NIER [37].

Fish species were grouped into four different trophic guilds (omnivores, piscivores, insectivores, and herbivores) and three different tolerance guilds (*i.e.*, tolerant species, intermediate species, and sensitive species) to evaluate how these traits were related to environmental differences. Tolerance and trophic guilds were defined according to the guidelines of the “National Surveys for Stream Ecosystem Health” in Korea [37]. A dataset consisting of 691 sites with fish species observed at more than two sampling sites were used for patterning and predicting fish communities. To reduce variation, fish density was natural-logarithm transformed after adding one to all data to avoid the problem of zero being undefined for logarithms.

### 2.2. Data Analysis

#### 2.2.1. Overall Procedure

**Figure 2:**
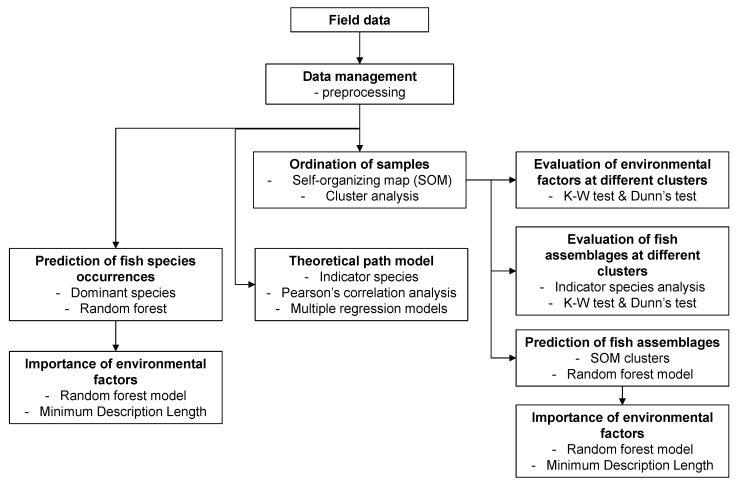
Overall data analyses procedures.

Data analyses were conducted with four different analytical methods: self-organizing map (SOM), indicator species analysis, random forest model, and theoretical path model: (1) SOM classified fish communities to several groups, (2) indicator species were defined in each group using indicator species analysis, (3) SOM groups were predicted with random forest model, (4) occurrences of selected species were predicted with random forest model, and (5) theoretical path model was used to evaluate the influence of environmental factors on the occurrence of indicator species. The overall data analyses procedures are given in Figure 2.

#### 2.2.2. Self-Organizing Map (SOM)

SOM was applied to characterize the distributional patterns of fish communities. SOMs average the dataset in weight vectors through the learning process while removing noise [39,40]. The weight vectors in SOM tend to approximate the probability density function of the input vector and provide the distributional pattern of each input variable [41]. SOM consists of an input layer and an output layer. Each layer is connected by connection intensities (weights). The input layer is formed by computation units (neurons) that receive input data, which are used to calculate the Euclidean distance between the weight vector and the input vector. The output layer consists of N output neurons on a two-dimensional hexagonal lattice. A map size of 126 (= 14 × 9) output neurons was chosen, which was determined by the heuristic equation [42]. Two criteria, the quantization error for resolution and the topographic error for topology preservation, were used to evaluate the map quality [41]. These error values were used as an indicator of the accuracy of the mapping at preserving topology [43].

After the learning process, the SOM units were classified based on a hierarchical cluster analysis using Ward’s linkage method with the Euclidean distance measure [44]. Based on the SOM weight after the SOM training, 25 relatively abundant species were examined to find indicative species for each cluster in SOM. We used the functions in the SOM toolbox [45] for training the SOM in Matlab version 6.1. A multi-response permutation procedure (MRPP), a nonparametric procedure for testing the differences between groups, was conducted using PC-ORD version 4.25 to evaluate the significance of the clusters [46].

#### 2.2.3. Random Forest Model

A random forest model [47] was applied to predict the occurrence of fish species and the patterns of fish communities as defined by the SOM, using different combinations of environmental variables, and to identify the determinant environmental factors contributing to the models. The random forest model is a non-parametric method for predicting and assessing the relationship between a large number of potential predictor variables and response variables [47]. Random forest models have several advantages compared to other statistical methods, such as high classification accuracy, a novel method of determining variable importance, and the ability to model complex interactions among predictor variables [48]. Therefore, the random forest model offers powerful alternatives to traditional parametric and semiparametric statistical methods for the analysis of ecological data.

The importance of environmental variables used in the random forest model was evaluated using the Minimum Description Length (MDL), which measures the quality of attributes as their ability to compress the data [49] To compare the relative importance of each environmental factor, values of MDL were rescaled to range from 0 to 100. Dichotomies (0 and 1) for SOM clusters were used to predict fish community patterns. We used a correct prediction rate (*i.e.*, the number of sites correctly predicted out of the total number of sites in the cluster) and Cohen’s kappa [50] as a measure of agreement. Cohen’s kappa ranges from 0 (completely random predictions) to 1 (perfect predictions). 

The random forest model was run with the *CORElearn* package [51] in the R statistical program (http://cran.r-project.org). Prior to running the random forest model, environmental variables, except land cover type, were transformed by the natural logarithm (x + 1) to reduce variation. 

#### 2.2.4. Indicator Species Analysis

To evaluate the indicator species in each SOM cluster, we applied indicator species analysis [52]. Indicator species were selected by an indicator value calculated as the product of its relative abundance and its relative frequency with ranges from 0 (no indication) to 100 (perfect indication) [53]. Monte Carlo tests were used to determine the significance of species indicator values. Indicator species analyses and Monte Carlo tests were carried out using PC-ORD version 4.25.

#### 2.2.5. Theoretical Path Model (TPM)

TPM was used to describe the directed dependencies among a set of variables at multiple spatial scales [54]. Path analysis [55,56,57] was used to decompose correlations into their direct and indirect components and to allow simple correlations among a set of variables to be partitioned according to a path model describing their causal relationships [58]. Path analysis generated diagrams representing the relationships between variables at different scales, their direct link, and its significance [59]. This included correlation coefficients from the Pearson’s correlation analysis and explanatory values (*R^2^*) for environmental variables from multiple regression models (MRMs). Environmental variables were placed into three groups (geo-hydrological factors, land cover types, physicochemical factors) with one response layer (indicator species). Only a single important indicator was employed for each SOM cluster in the TPM. The indicator species were selected using indicator species analysis. TPMs were conducted using Statistica software (StatSoft, Inc., version 7).

#### 2.2.6. Statistical Analysis

Spearman rank correlation coefficients were calculated among environmental variables. The Kruskal–Wallis test (K–W test) was used to compare the differences in environmental variables, community indices, and biological guilds, such as trophic and tolerance guilds, among clusters defined in the SOM. The nonparametric Dunn’s multiple comparisons test was used for *post hoc* comparisons. The K–W test and the Dunn’s test were conducted using the Statistica software.

## 3. Results

### 3.1. Fish Communities

A total of 128 fish species in 32 families were collected from 691 sites (Table 2), including 49 endemic species (representing 34.3% of the total fish abundance) and five exotic species (representing 3.3% of the total fish abundance). *Zacco platypus* (32.5%), *Z. koreanus* (11.6%), and *Pungtungia herzi* (4.4%) were the most abundant species and made up 48.6% of all the individuals collected. The Han River Watershed showed the highest species richness (96 species), followed by the Geum (69 species), Nakdong (67 species), Youngsan (59 species), and Seomjin (50 species) River Watersheds. The most common species in each of the five major watersheds was the same species that was the most common at the national scale, *Z. platypus*, but the second most common species was different in the Geum River Watershed (*Hemiculter eigenmanni*) and Youngsan River Watershed (*Z. temminckii*) from the other watersheds (*Z. koreanus*).

**Table 2:** Fish assemblage characteristics for different watersheds.

	Number of species			Dominant species	
Watershed	Total	Endemic	Exotic	1st	2nd
Han River	96	30	5	*Zacco* * platypus*	*Zacco* * koreanus*
Nakdong River	67	23	3	*Z. platypus*	*Z. koreanus*
Geum River	69	26	4	*Z. platypus*	*Hemiculter* * eigenmanni*
Youngsan River	59	18	3	*Z. platypus*	*Z. temminckii*
Seomjin River	50	19	3	*Z. platypus*	*Z. koreanus*
Total	128	49	5	*Z. platypus*	*Z. koreanus*

### 3.2. Relations between Environmental Factors

Altitude was significantly correlated with all environmental factors (Spearman rank correlation, *P* < 0.05). Forest area (*r* = 0.50, *P* < 0.01), slope (*r* = 0.44, *P* < 0.01), dry field area (*r* = 0.17, *P* < 0.01), and pH (*r* = 0.08, *P* < 0.05) showed positive correlations with altitude, while other factors showed negative correlations, especially water quality factors, except for pH (*r* < −0.10, *P* < 0.01) (Table 3). DFS was highly correlated with stream order (*r* = 0.86, *P* < 0.01), but both factors were not significantly correlated with land cover types except for forest area (*r* = −0.17 and −0.21, respectively, *P* < 0.01). Forest area was positively correlated with slope (*r* = 0.59, *P* < 0.01), altitude (*r* = 0.50, *P* < 0.01) and dry field area (*r* = 0.07, *P* < 0.05), and negatively correlated with geo-hydrological factors such as DFS (*r* = −0.17, *P* < 0.05). Meanwhile, TN and TP had significant correlations with all environmental factors except for hydrological factors and pH (*P* < 0.05).

### 3.3. Fish Assemblage Patterns

**Figure 3:**
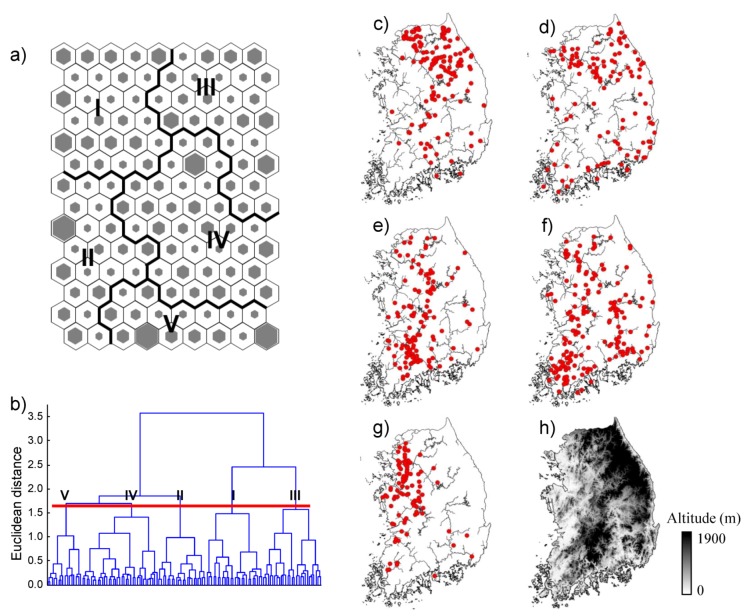
(**a**–**b**) Classification of 691 samples into five clusters (I–V) through the training of self-organizing map (SOM) and (**c**–**g**) the geographical distribution patterns of the sampling sites in five clusters, and (**h**) a digital elevation map (DEM) for comparing the relative elevation of each cluster.

Through the SOM learning process, the 691 sites were grouped into five clusters (I–V) according to the similarities of fish communities (Figure 3). The final quantization and topographic errors were 0.89 and 0.03, respectively, indicating a good training of the SOM. The clusters were significantly different in community composition (MRPP, A = 0.11, *P* < 0.001). The largest number of sites (181) was grouped into cluster IV, followed by cluster I (142), cluster III (138), cluster II (121), and cluster V (109). The number of sites in each cluster was visualized as the size of the hexagonal lattice in each SOM unit.

The distribution of sites in each cluster was highly related to the sites’ geographic locations (Figure 3(c–g)). For instance, most sites in cluster I were located in the northeastern part of the Han River Watershed, and the sites in cluster III were situated in a mountainous area of the Korean peninsula. Sites in cluster IV were widely distributed in the Youngsan and Nakdong Rivers. Most sites in cluster II were near the coast and the sites in cluster V were mainly in the tributaries of the Han River.

### 3.4. Differences in Environmental Variables

All environmental variables were significantly different among clusters (K–W test, *P* < 0.05) (Table 4). Altitude and slope were highest in cluster I, and lowest in cluster V (Dunn’s test, *P* < 0.05). Stream order was significantly lower in cluster II, and DFS in cluster III and IV was significantly greater than in the other clusters (Dunn’s test, *P* < 0.05). Cluster V showed high values for the proportion of urban area, but the lowest values for dry field area. Clusters I and II had a lower proportion of paddy fields than clusters III, IV, and V (Dunn’s test, *P* < 0.05).

The proportion of forest area decreased according to gradients of clusters from cluster I to cluster V (Dunn’s test, *P* < 0.05). The values of BOD, conductivity, and TP were significantly higher in cluster V, whereas cluster I showed the lowest values (Dunn’s test, *P* < 0.05). The concentration of TN was not significantly different among clusters except cluster V. Cluster C had a relatively low pH, and cluster I showed a significantly lower concentration of Chl-*a* than clusters II–IV.

**Table 3:** Spearman rank correlation coefficients between 14 environmental factors (* *P* < 0.05, ** *P* < 0.01).

Category	Variable	Geo-hydrological factors				Land cover types				Physicochemical factors				
		Altitude	Slope	DFS	Stream order	Urban	Forest	Paddy field	Dry field	pH	EC	BOD	TN	TP
Geo-hydrological factors	Slope	0.44 **												
	DFS	−0.17 **	−0.22 **											
	Stream order	−0.20 **	−0.26 **	0.86 **										
Land cover types	Urban	−0.21 **	−0.23 **	−0.05	−0.01									
	Forest	0.50 **	0.59 **	−0.17 **	−0.21 **	−0.42 **								
	Paddy field	−0.29 **	−0.35 **	0.02	0.02	−0.16 **	−0.45 **							
	Dry field	0.17 **	0.07	0.04	0.05	0.02	0.07 *	−0.17 **						
Physicochemical factors	pH	0.08 *	−0.03	0.26 **	0.25 **	−0.10 **	0.03	−0.04	0.08 *					
	EC	−0.45 **	−0.31 **	0.09 *	0.21 **	0.22 **	−0.41 **	0.17 **	−0.06	0.08 *				
	BOD	−0.37 **	−0.30 **	0.07	0.13 **	0.21 **	−0.39 **	0.20 **	−0.12 **	0.02	0.55 **			
	TN	−0.28 **	−0.18 **	−0.07	−0.03	0.21 **	−0.27 **	0.09 *	−0.08 *	−0.02	0.46 **	0.63 **		
	TP	−0.47 **	−0.29 **	−0.01	0.05	0.24 **	−0.43 **	0.21 **	−0.12 **	−0.01	0.56 **	0.74 **	0.63 **	
	Chl−*a*	−0.11 **	−0.13 **	0.14 **	0.16 **	0.12 **	−0.17 **	0.02	−0.05	0.04	0.23 **	0.37 **	0.22 **	0.28 **

**Table 4:** Differences in environmental variables among the five clusters defined by the SOM. The number indicates mean (1st quartile–3rd quartile) in each clusters. Different letters in each row indicate significant differences for that variable among the clusters based on Dunn’s multiple comparison tests (*P* < 0.05).

Category	Variable			SOM cluster		
		I	II	III	IV	V
Geo-hydrological factors	Altitude (m)	210.3 (91–281) ^a^	132.7 (15–166)^ c^	106.3 (44–146)^ b^	55.6 (19–76) ^c, d^	41.2 (16–45)^ d^
	Slope (%)	23.1 (4–36)^ a^	17 (1.5–28)^ b^	10.4 (1–15)^ b^	11.2 (1–12)^ b^	4.7 (1–5)^ c^
	DFS (km)	33.8 (12.1–40.8)^ b^	56.2 (5.3–34.4)^ c^	55.1 (16.7–69.5)^ a^	71.1 (14.9–72.6)^ a^	32 (11.4–32.8)^ bc^
	Stream Order	5 (4–6)^ bc^	5 (3–5) ^ c^	6 (5–6)^ a^	6 (5–7)^ a^	5 (4–6)^ ab^
Land cover types	Urban (%)	11.5 (1.8–16.6)^ b^	18.1 (1.3–22.6)^ b^	10.9 (1–14.3)^ b^	14.9 (2.3–15.8)^ b^	34.1 (6–62.9)^ a^
	Forest (%)	47.1 (19.6–76.2)^ a^	39 (5.1–68.8)^ ab^	31.7 (6.9–53.2)^ b^	22.8 (0–38.5) ^c^	8 (0–7.6) ^d^
	Paddy field (%)	18.3 (0–30.6)^ b^	20.3 (0–34.9)^ b^	32.2 (10.8–51)^ a^	31.6 (4.3–49.2)^ a^	35.1 (1.1–63.4)^ a^
	Dry field (%)	15.6 (3–22.9)^ a^	12.5 (0–20.6)^ a^	11.9 (0.8–17.2)^ a^	12.3 (0.8–17.2)^ a^	7.9 (0–14)^ b^
Physico-chemical factors	pH	8.3 (7.6–9)^ a^	8.1 (7.3–8.9)^ a, b^	8.2 (7.6–8.7)^ a^	8.2 (7.5–8.8)^ a^	7.9 (7.4–8.4)^ b^
	EC (*μ*S/cm)	181.4 (82.9–204.8) ^d^	285.7 (106.0–398.0)^ b, c^	231.4 (133.2–284.9)^ c^	311.9 (171.6–377.5)^ b^	471 (302–542.2)^ a^
	BOD (mg/L)	1.6 (1–2)^ c^	2.5 (1.2–3.2)^ b^	2.7 (1.3–3.4)^ b^	2.7 (1.4–3.4)^ b^	5.6 (3.3–7.2)^ a^
	TN (mg/L)	2 (1.3–2.3)^ b^	2.7 (1.3–3.3)^ b^	2.2 (1–2.9)^ b^	2.5 (1.2–3.3)^ b^	5.7 (3.4–6.7)^ a^
	TP (mg/L)	0.03 (0.01–0.03)^ c^	0.11 (0.02–0.11)^ b^	0.07 (0.02–0.08)^ b^	0.11 (0.02–0.14)^ b^	0.3 (0.1–0.4)^ a^
	Chlorophyll- *a* (*μ*g/L)	2.3 (0.4–2.6)^ c^	5.6 (0.5–5.4)^ ab^	5.8 (1–7)^ a^	5.4 (0.4–4.7)^ b^	7.8 (1.4–4.5)^ a^

### 3.5. Characteristics of Fish Assemblages

**Figure 4:**
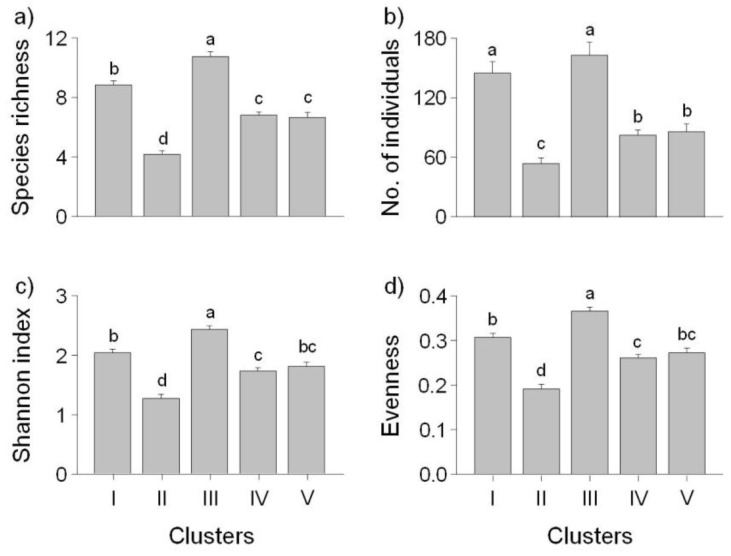
Mean differences (±S.E.) in community indices for five clusters defined by the self-organizing map (SOM): (**a**) species richness, (**b**) number of individuals, (**c**) Shannon index, and (**d**) evenness. Different letters indicate significant differences between the clusters based on Dunn’s test after a Kruskal–Wallis test (*P* < 0.05).

**Figure 5:**
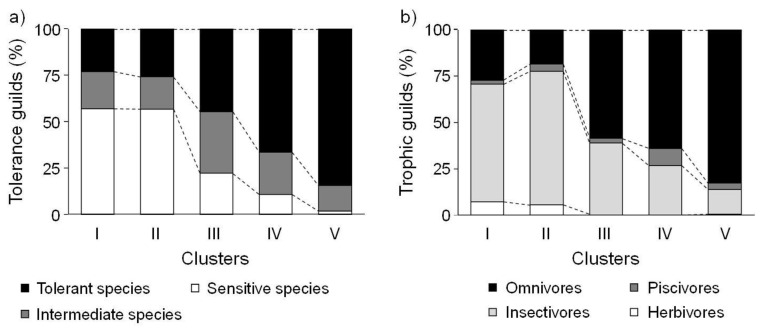
(**a**) Differences in tolerance and (**b**) trophic guilds for five clusters defined by the self-organizing map (SOM).

Community indices, tolerance guilds, and trophic guilds were significantly different among the five SOM clusters (K–W test, *P* < 0.05) (Figure 4). Species richness, the number of individuals, the Shannon index, and evenness showed similar patterns. Community indices, except for the number of individuals, were significantly higher in cluster III, and the lowest in cluster II (Dunn’s test, *P* < 0.05). The ratio of insectivores to herbivores among the feeding guilds was relatively high in cluster I and was the lowest in cluster V (Figure 5). 

The proportion of herbivores showed a similar pattern to that of insectivores, and was significantly higher in cluster I than in all other clusters except cluster II (Dunn’s test, *P* < 0.05). The proportion of omnivores was higher in cluster V, whereas that of piscivores was higher in cluster IV (Dunn’s test, *P* < 0.05).

### 3.6. Prediction of Fish Assemblages

**Figure 6:**
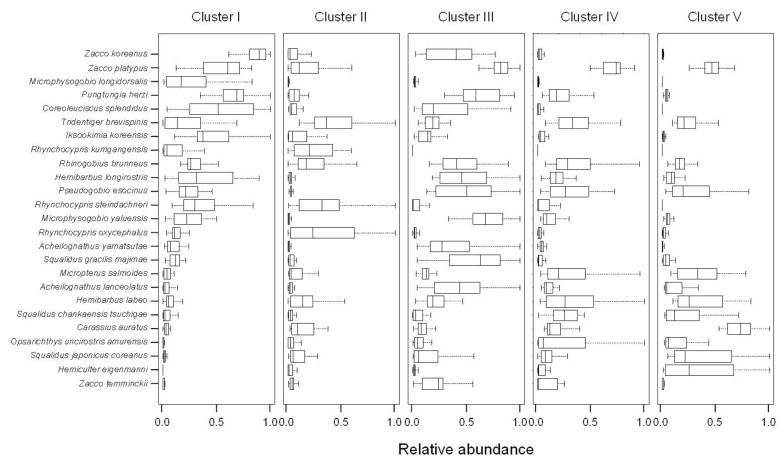
Relative abundance of 25 dominant species in difference clusters. Values were obtained from weight vectors of the trained self-organizing map (SOM) (boxplot: - median, 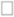 25-75% percentiles, 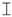 non-outlier range).

All five clusters were well predicted by random forest models (prediction accuracy > 0.75) according to several different combinations of environmental factors across multiple spatial scales (Table 5). When all 14 environmental factors were considered as independent variables, the prediction accuracy was the highest (>0.85). The prediction accuracies were low when only the four land cover types were used. Cluster I had higher abundances of *Z. koreanus*, *Coreoleuciscus splendidus*, and *Tridentiger brevispinis*, and cluster II had a high abundance of *Rhynchocypris oxycephalus* (Figure 6). Cluster IV was characterized by a relatively high abundance of *T. brevispinis* and *Z. platypus*, whereas cluster V was represented by *Carassius auratus* and *H. eigenmanni*.

**Table 5:** Prediction for each cluster using the random forest model with different combinations of environmental variables.

Cluster		Geo	Land	WQI	Geo + land	Geo + WQI	Land + WQI	Total
I	Prediction rate	0.89	0.84	0.89	0.89	0.91	0.89	0.92
	Kappa (*k*)	0.60	0.36	0.62	0.62	0.68	0.62	0.74
II	Prediction rate	0.87	0.83	0.85	0.88	0.89	0.84	0.89
	Kappa (*k*)	0.41	0.00	0.16	0.47	0.49	0.11	0.51
III	Prediction rate	0.85	0.81	0.86	0.86	0.88	0.88	0.88
	Kappa (*k*)	0.37	0.07	0.41	0.42	0.51	0.50	0.53
IV	Prediction rate	0.80	0.76	0.77	0.83	0.86	0.81	0.88
	Kappa (*k*)	0.32	0.14	0.18	0.44	0.58	0.35	0.63
V	Prediction rate	0.88	0.88	0.93	0.92	0.93	0.94	0.94
	Kappa (*k*)	0.32	0.40	0.69	0.60	0.69	0.74	0.74

Geo: geo-hydrological factors; Land: land cover types; WQI: physicochemical factors; Total: all 14 environmental factors.

The occurrences of 15 indicator species (Table 6) were well predicted with 14 environmental factors using the random forest model (accuracy rate >0.85). *H. eigenmanni* and *Sarcocheilichthys variegatus* displayed the highest prediction accuracy (0.96 and 0.95, respectively). Altitude and DFS were the most important variables for the prediction of fish occurrence. Forest area, stream order, TP, and conductivity were also relatively important for the prediction of fish occurrence.

**Figure 7:**
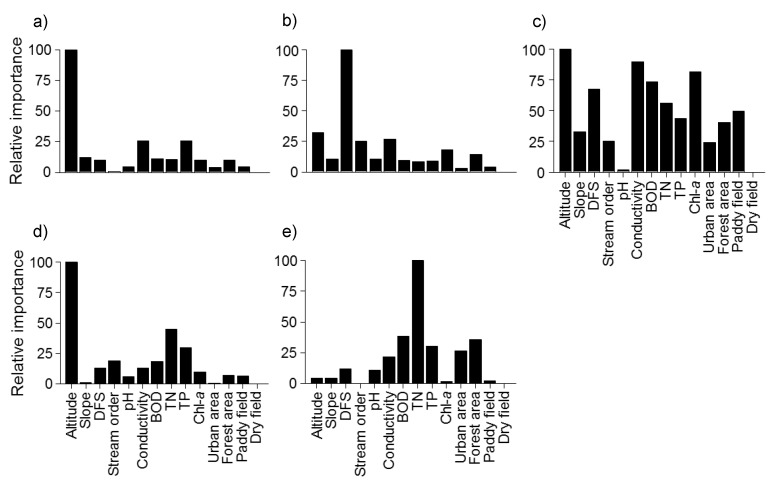
Importance of environmental variables in each cluster defined in SOM using a random forest model.

Altitude was the most important variable for the prediction of fish community patterns in clusters I, III, and IV (Figure 7); while DFS was the most important variable for cluster II and TN was the most important for cluster V.

**Table 6:** Prediction of fish species and evaluation of variable importance using the random forest model.

Species	Prediction rate	Kappa (*k*)	Important factors (MDL)		Cluster	
			1st	2nd	Indicator	Dominance
*Zacco* * platypus*	0.88	0.63	DFS (100)	Altitude (45.7)	III	III
*Liobagrus* * andersoni*	0.93	0.13	Urban (100)	pH (97.1)	I	I
*Sarcocheilichthys* * variegatus*	0.95	0.55	DFS (100)	Conductivity (61.3)	III	III
*Iksookimia* * koreensis*	0.92	0.66	Altitude (100)	DFS (44.3)	I	I
*Zacco* * koreanus*	0.90	0.76	Altitude (100)	TP (42.6)	I	I
*Cyprinus* * carpio*	0.93	0.68	TN (100)	TP (88.2)	V	V
*Coreoleuciscus* * splendidus*	0.94	0.82	Altitude (100)	DFS (78.9)	I	I
*Koreocobitis* * rotundicaudata*	0.92	0.31	Urban (100)	DFS (68.4)	I	I
*Carassius* * auratus*	0.89	0.75	TP (100)	Forest (57.2)	V	V
*Pseudogobio* * esocinus*	0.91	0.81	DFS (100)	Stream order (60.6)	III	III
*Microphysogobio* * longidorsalis*	0.95	0.58	Altitude (100)	DFS (78.4)	I	I
*Microphysogobio* * yaluensis*	0.90	0.65	Altitude (100)	BOD (47.3)	III	III
*Pungtungia* * herzi*	0.90	0.79	Altitude (100)	Conductivity (80.0)	I, III	III
*Coreoperca* * herzi*	0.93	0.75	DFS (100)	TP (66.0)	I	I
*Squalidus* * gracilis*	0.89	0.54	Altitude (100)	Paddy field (79.1)	III	III
*Rhynchocypris* * oxycephalus*	0.93	0.62	DFS (100)	Altitude (65.6)	-	II
*Hemiculter* * eigenmanni*	0.96	0.62	Altitude (100)	Forest (74.3)	-	V
*Tridentiger* * brevispinis*	0.92	0.22	DFS (100)	Stream order (36.4)	-	I

### 3.7. Theoretical Path Model

**Figure 8:**
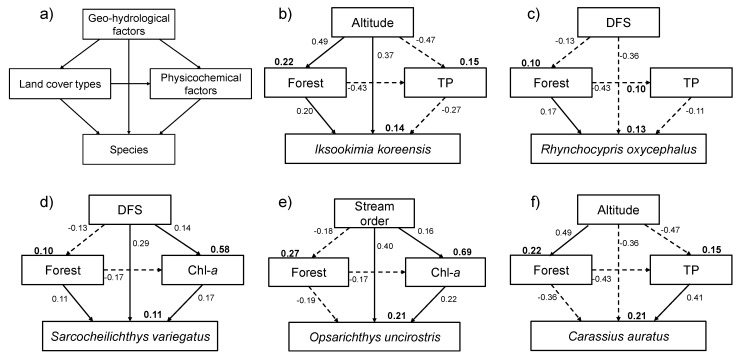
Path diagrams to estimate the relationship between environmental variables in 3 different subgroups (geo-hydrology, land cover type, physicochemistry) and prevalence of major species in each cluster by the self-organizing map (SOM). (**a**) Structure of path diagram, (**b**) *Iksookimia koreensis* (major species in cluster I), (**c**) *Rhynchocypris oxycephalus* (major species in cluster II), (**d**) *Sarcocheilichthys variegatus* (major species in cluster III), (**e**) *Opsariichthys uncirostris* (major species in cluster IV), and (**f**) *Carassius auratus* (major species in cluster V). Arrows represent directed relationships. Solid lines indicate significantly (*P* < 0.05) positive relationships and broken lines indicate significantly (*P* < 0.05) negative relationships. *R^2^* values by linear multiple regression are shown in bold.

Different species showed different responses to their environment across multiple spatial scales. Among geo-hydrological factors, altitude was positively correlated with *Iksookimia koreensis*, which was an indicator species of cluster I, whereas *Carassius auratus*, which was an indicator species of cluster V, displayed a negative correlation (*r* = 0.37 and *r* = −0.36, respectively, *P* < 0.05) (Figure 8). *Rhynchocypris oxycephalus* and *S. variegatus* showed a highly significant correlation with DFS (*r* = −0.36 and *r* = 0.29, respectively, *P* < 0.05). At the local scale, the presence of *S. variegatus* and *Opsariichthys uncirostris* was positively correlated with the concentration of Chl-*a* (*r* = 0.17 and *r* = 0.22, respectively, *P* < 0.05), whereas other species exhibited a significant relationship with TP (*r* = −0.27 *in I. koreensis*, *r* = −0.11 in *R. oxycephalus*, and *r* = 0.41 in *C. auratus*, *P* < 0.05). 

Forest area was the most important land cover variable for evaluating the relationship between the appearance of major species in each cluster and environmental variables. Forest area showed a negative correlation with *O. uncirostris* and *C. auratus* (*r* = −0.19 and *r* = −0.36, respectively, *P* < 0.05), while the other three major species exhibited positive correlations. 

## 4. Discussion

The distribution and abundance of fish communities were characterized with environmental variables across multiple spatial scales using SOM, random forest, and theoretical path models. In this study, we characterized how Korean fish assemblages on a national scale react to changes in the modified longitudinal gradient with various environmental variables at multiple spatial scales, and presented the importance of altitude, DFS, and urban areas for predicting fish community patterns and the occurrence of fish species. These results could provide necessary information for managing fish assemblages and the relationships between changes in fish assemblage and environmental variables.

SOM revealed differences among fish communities, reflecting environmental gradients such as the longitudinal gradient from upstream to downstream, and differences in land cover, water quality, *etc.* For example, sites in cluster I were from small streams (25.7% of the streams less than third order with high altitude and short DFS), whereas most sites in cluster IV were located further downstream (39.9% of streams were greater than the seventh order with low altitude and long DFS). Species richness and abundance were significantly lower at downstream sites, and high values were found at mid-stream sites. However, previous studies have reported trends where the lowest species richness and abundance is found in headwater streams and the highest levels are downstream at low altitudes [13,60,61,62,63]. These studies highlighted the importance of habitat size, because the larger area supported higher species richness and abundance. However, this concept was primarily applied to areas without disturbances. In the current study, the sampling sites showed a wide range of disturbances but general longitudinal gradients of fish species richness were observed after excluding severely polluted sites from the analysis. Oberdorff *et al.* [64] support our findings that species richness reached the maximum in midsize rivers, and then decreased in large rivers. The proportion of forest area decreased downstream, whereas agricultural and urban areas increased, creating an increase in nutrient and pollutant inputs to streams [65]. The moderate increase of nutrients in the middle stream led to increased species richness, while high nutrients may have reduced the species richness. This supports the intermediate disturbance hypothesis [66,67,68].

Trophic guilds as well as species richness changed along the upstream-downstream gradient. This result supported the River Continuum Concept [69]. The proportions of herbivores and insectivores were significantly higher further upstream than downstream, whereas the proportion of omnivores was relatively high downstream (Figure 5). The trophic composition of the fish communities was induced by the available food resources [64]. Lowe-McConnell [70] and Rahel and Hubert [71] reported similar results; headwater streams had higher proportions of insectivorous species, while omnivores were more common in large rivers. These gradients in longitudinal distribution were found at the species level (Figure 6). Insectivores such as *Iksookimia koreensis*, *C. splendidus*, and *Z. koreanus* were mainly distributed in cluster I, and their relative abundance gradually decrease towards clusters III and IV. Piscivores, such as *O. uncirostris*, showed relatively high abundance in cluster IV, and gradually decreased toward cluster I.

Urbanization was correlated with low fish abundance and richness and urban sites were dominated by disturbance-tolerant species [72]. Urbanization can lead to high concentrations of TP and TN [73], however, fish diversity and abundances in urban catchments have been found to be dramatically lower than in forested catchments [74,75,76]. This relationship indicates that urbanization can exert a major influence on water quality, habitat, and biological assemblages [65]. Similarly, agricultural exploitation can also influence aquatic organisms and their environments. Many studies have reported that agricultural activities degrade water quality, affect both riparian and stream habitat quality, and alter water flow [65]. Fish and macroinvertebrate biodiversity has been documented to decrease with a greater percentage of agricultural land [77,78,79].

Fish assemblages can be influenced by changes in environmental variables such as physical habitat and land use [80,81,82,83]. Stream gradient, stream order, hydrologic regime, and channel morphology were highly correlated with species richness [84,85,86]. Joy and Death [87] showed that altitude and distance from the coast were important in a model predicting regional freshwater fish occurrence in the Manawatu–Wanganui region of New Zealand. He *et al.* [27] also stated that altitude and stream length played important roles in driving the observed endemic fish assemblage structure. Altitude and DFS were also important variables for the prediction of fish community patterns in this study (Figure 7). Especially, altitude was the most important variable for the prediction of fish community pattern in Clusters I, III and IV, an indication of longitudinal gradients.

Altitude and DFS were the most important factors in 11 of the 15 indicator species. These 11 were indicator species for clusters I and III, which had relatively high altitude. *Coreoperca herzi* was an indicator species for cluster I, and DFS and TP were relatively important for predicting the occurrence of *C. herzi*. Samples in cluster I were located in upstream locations with a short DFS and a low concentration of TP (Table 4). Urban land cover was the most important variable for predicting the distribution of *Liobagrus andersoni* and *Koreocobitis rotundicaudata*, which were indicator species of cluster I. Changes in land use can affect assemblage composition, and lead to changes in the contaminant level of streams [88]. TN and TP were the most important variables for predicting the distribution of *Cyprinus carpio* and *C. auratus*, which were indicator species for cluster V.

Similarly, the theoretical path model described different responses of species to their environment at multiple spatial scales (Figure 8). Geographical attributes persist over a relatively long time and influence the development and selection of species’ life history and behavioral traits [89]; and the surrounding conditions (e.g., slope and stream order) of a stream can also directly and indirectly affect stream habitats [1,90]. The theoretical path model showed significant correlations between geo-hydrological factors, land cover types, and physicochemical factors. Among land cover types, forest area displayed the highest correlation with five dominant species, and Chl-*a* and TP were the most important physicochemical factors for explaining species occurrences, indicating the importance of water quality in micro-habitat condition. Li *et al.* [1] reported similar results on benthic macro-invertebrates in the same study area. There are many reports of a strong correlation between geographical location and stream communities [91,92] and of the importance of altitude [3,93]. 

The random forest model is a non-parametric method for predicting and assessing the relationship between a large number of potential predictor variables and response variables [47]. Cutler *et al.* [48] reported that the random forest model demonstrated its learning and predicative power as well as its explanatory capacities by presenting a high capability for modeling ecological problems involving non-linear relationships between data. Random forest models have several advantages compared to other statistical methods, such as high classification accuracy, a novel method of determining variable importance, and the ability to model complex interactions among predictor variables [48]. Therefore, the random forest model offers powerful alternatives to traditional parametric and semiparametric statistical methods for the analysis of ecological data. In addition, He *et al.* [27] showed that mixed models that included both land cover and river characteristic variables were more powerful at explaining the endemic fish distribution patterns in the upper Yangtze River, similar to our results. In our study, the random forest model was more powerful for predicting fish community patterns using all 14 environmental factors than models using either a single variable or another combination of environmental variables (Table 5).

Many studies have been conducted on the relationships between changes in fish community structure and environmental variables [71,82,83], and most studies have considered some environmental variables such as physical habitat and land use at the local or watershed scale [94]. Although the distribution and abundance of species are closely linked to small-scale habitat availability [95], they are also influenced by variables at larger spatial scales [91]. Regional variables may operate as “filters” constraining species at lower scales through selective habitat forces [22]. Consequently, preservation and conservation strategies for maintaining stream integrity will be more effective if they are treated as a part of landscape development rather than an isolated entity [1]. Future studies to benefit conservation and management may consider the influences of global processes on biodiversity, the interactions between these three spatial scales, and the effects of global warming on fish communities. 

## 5. Conclusions

The relationships between the distribution and abundance of fish communities and environmental variables at multiple spatial scales were evaluated using SOM, random forest, and theoretical path models. The SOM explored differences among fish communities, reflecting environmental gradients, such as a longitudinal gradient from upstream to downstream, and differences in land cover types and water quality. The random forest model for predicting fish community patterns that used all 14 environmental variables was more powerful than a model using any single variable or other combination of environmental variables, and the random forest model was effective at predicting the occurrence of species and evaluating the contribution of environmental variables to that prediction. The theoretical path model described the responses of different species to their environment at multiple spatial scales, showing the importance of altitude in geo-hydrological factors, forest cover types, and water quality factors to fish assemblages. 

## References

[1] Li F., Chung N., Bae M.-J., Kwon Y.-S., Park Y.-S. (2012). Relationships between stream macroinvertebrates and environmental variables at multiple spatial scales. Freshwater Biol..

[2] Levin S.A. (1992). The problem of pattern and scale in ecology. Ecology.

[3] Townsend C.R., Dolédec S., Norris R., Peacock K., Arbuckle C. (2003). The influence of scale and geography on relationships between stream community composition and landscape variables: Description and prediction. Freshwater Biol..

[4] Mykrä H., Heino J., Muotka T. (2007). Scale-related patterns in the spatial and environmental components of stream macroinvertebrate assemblage variation. Global Ecol. Biogeogr..

[5] Carpenter S.R., Kitchell J.F., Hodgson J.R. (1985). Cascading trophic interactions and lake productivity. BioScience.

[6] Power M.E., Matthews W.J., Stewart A.J. (1985). Grazing minnows, piscivorous bass, and stream algae: Ddynamics of a strong interaction. Ecology.

[7] Wootton J.T., Power M.E. (1993). Productivity, consumers, and the structure of a river food chain. Proc. Natl. Acad. Sci. USA.

[8] Karr J.R., Freemark K.E., Pickett S.T.A., White P.S. (1985). Disturbance and Vertebrates: An Integrative Perspective. Ecology of Natural Disturbance and Patch Dynamics.

[9] McCauley R.W. (1990). Determining the health of fish communities—Parallels with human medicine. J. Great Lakes Res..

[10] Kouamélan E.P., Teugels G.G., N’Douba V., Bi G.G., Koné T. (2003). Fish diversity and its relationships with environmental variables in a West African basin. Hydrobiologia.

[11] Beechie T.J., Sibley T.H. (1997). Relationships between channel characteristics, woody debris, and fish habitat in northwestern Washington streams. T. Am. Fish. Soc..

[12] Allan J.D. (1995). Reference. Stream Ecology: Structure and Function of Running Waters.

[13] Park Y.S., Grenouillet G., Esperance B., Lek S. (2006). Stream fish assemblages and basin land cover in a river network. Sci. Total Environ..

[14] Burton G.W., Odum E.P. (1945). The distribution of stream fish in the vicinity of mountain lake, Virginia. Ecology.

[15] Torgersen C.E., Baxter C.V., Li H.W., McIntosh B.A. (2006). Landscape influences on longitudinal patterns of river fishes: Spatially continuous analysis of fish-habitat relationships. Amer. Fish. Soc..

[16] Kuehne R.A. (1962). A classification of streams, illustrated by fish distribution in an eastern Kentucky creek. Ecology.

[17] Harrel R.C., Davis B.J., Dorris T.C. (1967). Stream order and species diversity of fishes in an intermittent Oklahoma stream. Am. Midl. Nat..

[18] Paller M.H. (1994). Relationships between fish assemblage structure and stream order in South Carolina plain streams. Am. Fish. Soc..

[19] Whiteside B.G., McNatt R.M. (1972). Fish species diversity in relation to stream order and physicochemical conditions in the Plum Creek drainage basin. Am. Midl. Nat..

[20] Schofield C.L., Driscoll C.T. (1987). Fish species distribution in relation to water quality gradients in the North Branch of the Moose River Basin. Biogeochemistry.

[21] Tonn W.M., Magnuson J.J., Rask M., Toivonen J. (1990). Intercontinental comparison of small-lake fish assemblages: The balance between local and regional processes. Am. Nat..

[22] Poff N.L. (1997). Landscape filters and species traits: Towards mechanistic understanding and prediction in stream ecology. J. N. Am. Benthol. Soc..

[23] Gregory S.V., Swanson F.J., McKee W.A., Cummins K.W. (1991). An ecosystem perspective of riparian zones. BioScience.

[24] Townsend C.R. (1996). Invasion biology and ecological impacts of brown trout Salmotrutta in New Zealand. Biol. Conserv..

[25] Gevrey M., Park Y.S., Oberdorff T., Lek S., Lek S., Scardi M., Verdonschot P.F.M., Desy J.P., Park Y.S. (2005). Predicting Fish Assemblages in France and Evaluating the Influence of Their Environmental Variables. Modelling Community Structure in Freshwater Ecosystems.

[26] Park Y.S., Oberdorff T., Lek S., Lek S., Scardi M., Verdonschot P.F.M., Desy J.P., Park Y.S. (2005). Patterning Riverine Fish Assemblages Using An Unsupervised Neural Network. Modelling Community Structure in Freshwater Ecosystems.

[27] He Y., Wang J., Lek-Ang S., Lek S. (2011). Predicting assemblages and species richness of endemic fish in the upper Yangtze River. Sci. Total Environ..

[28] Scardi M., Cataudella S., Ciccotti E., Di Dato P., Maio G., Marconato E., Salviati S., Tancioni L., Turin P., Zanetti M., Lek S., Scardi M., Verdonschot P.F.M., Desy J.P., Park Y.S. (2005). Optimisation of Artificial Neural Networks for Predicting Fish Assemblages in Rivers. ModellingCommunity Structure in Freshwater Ecosystems.

[29] Grenouillet G., Pont D., Hérissé C. (2004). Within-basin fish assemblage structure: The relative influence of habitat *versus* stream spatial position on local species richness. Can. J. Fish. Aquat. Sci..

[30] Marsh-Matthews E., Matthews W.J. (2000). Geographic, terrestrial and aquatic factors: Which most influence the structure of stream fish assemblages in the midwestern United States?. Ecol. Freshw. Fish..

[31] D’Ambrosio J.L., Williams L.R., Witter J.D., Ward A. (2009). Effects of geomorphology, habitat, and spatial location on fish assemblages in a watershed in Ohio, USA. Environ. Monit. Assess..

[32] Hayes J.W., Leathwick J.R., Hanchet S.M. (1989). Fish distribution patterns and their association with environmental factors in the Mokau River catchment, New Zealand. New Zeal. J. Mar. Fresh..

[33] Jowett I.G., Richardson J. (2003). Fish communities in New Zealand rivers and their relationship to environmental variables. New. Zeal. J. Mar. Fresh..

[34] Lee J.H., Han J.-H., Kumar H.K., Choi J.-K., Byeon H.K., Choi J.S., Kim J.-K., Jang M.-H., Park H.-K., An K.-G. (2011). National-level integrative ecological health assessments based on the index of biological integrity, water quality, and qualitative habitat evaluation index, in Korean rivers. Ann. Limnol. Int. J. Lim..

[35] Yoon J.-D., Kim J.-H., Byeon M.-S., Yang H.-J., Park J.-Y., Shim J.-H., Song H.-B.,  Yang H., Jang M.-H. (2011). Distribution patterns of fish communities with respect to environmental gradients in Korean streams. Ann. Limnol. Int. J. Lim..

[36] Fu C. (2003). Potential impacts of human-induced land cover change on East Asia monsoon. Global Planet. Change.

[37] The Ministry of Environment/ National Institute of Environmental Research, Korea (2008). The Survey and Evaluation of Aquatic Ecosystem Health in Korea.

[38] Eaton A.D., Clesceri L.S., Rice E.W., Greenberg A.E., Franson M.A.H. (2005). Standard Methods for the Examination of Water and Wastewater.

[39] Vesanto J., Himberg J., Siponen M., Simula O. Enhansing SOM Based Data Visualization. Proceedings of the 5th International Conference of Soft computing and information/Intelligent Systems (IIZUKA’98).

[40] Kohonen T. (2001). Reference. Self-Organizing Maps.

[41] Park Y.S., Céréghino R., Compin A., Lek S. (2003). Applications of artificial neural networks for patterning and predicting aquatic insect species richness in running waters. Ecol. Model..

[42] Vesanto J., Alhoniemi R. (2000). Clustering of the self-organizing map. IEEE T. Neural Networ..

[43] Céréghinoa R., Park Y.S. (2009). Review of the Self-Organizing Map (SOM) approach in water resources: commentary. Environ. Modell. Softw..

[44] Legendre P., Legendre L. (1998). Reference. Numerical Ecology.

[45] Alhoniemi E., Himberg J., Parhankangas J., Vesanto J. SOM Toolbox. http://www.cis.hut.fi/projects/somtoolbox.

[46] Mielke E.W., Berry K.J., Johnson E.S. (1976). Multiresponse permutation procedures for a priori classifications. Commun. Stat. Theor. M..

[47] Breiman L. (2001). Random forests. Mach. Learn..

[48] Cutler R.D., Edwards T.C., Beard K.H., Cutler A., Hess K.T., Gibson J., Lawler J.J. (2007). Random forests for classification in ecology. Ecology.

[49] Robnik-Sikonja M. (2004). Improving random forests. Mach. Learn..

[50] Cohen J. (1960). A coefficient of agreement for nominal scales. Educ. Psychol. Meas..

[51] Robnik-Sikonja M., Savicky P. CORElearn—Classification, Regression, Feature Evaluation and Ordinal Evaluation. The R Project for Statistical Computing, 2012. http://www.r-project.org.

[52] Dufrêne M., Legendre P. (1997). Species assemblages and indicator species: The need for a flexible asymmetrical approach. Ecol. Monogr..

[53] Peterson W.T., Keister J.E. (2003). Interannual variability in copepod community composition at a coastal station in the northern California Current: A multivariate approach. Deep Sea Res..

[54] Donovan R. (1984). Path analysis of a theoretical model of persistence in higher education among low-income Black youth. Res. High. Educ..

[55] Wright S. (1934). The method of path coefficients. Ann. Math. Statist..

[56] Mitchell R.J. (1992). Testing evolutionary and ecological hypotheses using path analysis and structural equation. Funct. Ecol..

[57] Shipley B. (1997). Explanatory path analysis with applications in ecology and evolution. Am. Nat..

[58] Pittman S.J., McAlpine C.A., Pittman K.M. (2004). Linking fish and prawns to their environment: A hierarchical landscape approach. Mar. Ecol. Prog. Ser..

[59] Mellin C., Andréfouët S., Kulbicki M., Dalleau M., Vigliola L. (2009). Remote sensing and fish–habitat relationships in coral reef ecosystems: Review and pathways for multi-scale hierarchical research. Mar. Pollut. Bull..

[60] Horwitz R.J. (1978). Temporal variability patterns and the distribution patterns of stream fishes. Ecol. Monogr..

[61] Penczak T., Mann R.H.K. (1990). The impact of stream order on fish population in the Pilica drainage basin, Poland. Pol. Arch. Hydrobiol..

[62] Schlosser I.J. (1990). Environmental variation, life history attributes, and community structure in stream fishes: Implications for environmental management and assessment. Environ. Mange..

[63] Oberdorff T., Pont D., Hugueny B., Chessel D. (2001). A probabilistic model characterizing fish assemblages of French rivers: A framework for environmental assessment. Freshwater Biol..

[64] Oberdorff T., Gilbert E., Lucchetta J.C. (1993). Patterns of fish species richness in the Seine River basin, France. Hydrobiologia.

[65] Allan J.D. (2004). Landscapes and river scapes: The influence of land use on stream ecosystems. Annu. Rev. Ecol. Evol. Syst..

[66] Grime J.P. (1973). Competitive exclusion in herbaceous vegetation. Nature.

[67] Horn H.S., Cody M.L., Diamond J.M. (1975). Markovian Processes of Forest Succession. Ecology and Evolution of Communities.

[68] Connell J.H. (1978). Diversity in tropical rain forests and coral reefs. Science.

[69] Vannote R.L., Minshall G.W., Cummins K.W., Sedell J.R., Cushing C.E. (1980). The river continuum concept. Can. J. Fish. Aqua. Sci..

[70] Lowe-McConnell R.H. (1975). Reference. Fish Communities in Tropical Freshwaters.

[71] Rahel F.J., Hubert W.A. (1991). Fish assemblages and habitat gradients in a Rocky Mountain-Great Plains stream: Biotic zonation and additive patterns of community change. Trans. Am. Fish. Sot..

[72] Morgan R.P., Cushman S.F. (2005). Urbanization effects on stream fish assemblages in Maryland, USA. J. N. Am. Benthol. Soc..

[73] Paul M.J., Meyer J.L. (2001). Streams in the urban landscape. Annu. Rev. Ecol. Syst..

[74] Scott J.B., Steward C.R., Stober Q.J. (1986). Effects of urban development on fish population dynamics in Kelsey Creek, Washington. Trans. Am. Fish. Soc..

[75] Weaver L.A., Garman G.C. (1994). Urbanization of a watershed and historical changes in a stream fish assemblage. T. Am. Fish. Soc..

[76] Lenat D.R., Crawford J.K. (1994). Effects of land use on water quality and aquatic biota of three North Carolina piedmont streams. Hydrobiologia.

[77] Genito D., Gburek W.J., Sharpley A.N. (2002). Response of stream macroinvertebrates to agricultural land cover in a small watershed. J. Freshwater Ecol..

[78] Wang L., Lyons J., Kanehl P., Gatti R. (1997). Influences of watershed land use on habitat quality and biotic integrity in Wisconsin streams. Fisheries.

[79] Harding J.S., Benfield E.F., Bolstad P.V., Helfman G.S., Jones E.B.D. (1998). Stream biodiversity; The ghost of land use past. Proc. Natl. Acad. Sci. USA.

[80] Karr J.R., Toth L.A., Dudley D.R. (1985). Fish communities of midwestern rivers: A history of degradation. BioScience.

[81] Roth N.E., Allan J.D., Erickson D.L. (1996). Landscape influences on stream biotic integrity assessed at multiple spatial scales. LandscapeEcol..

[82] Hughes R.M., Wang L., Seelbach P.W. (2006). Reference. Landscapes Influences on Stream Habitats and Biological Assemblages.

[83] McClelland M.A., Pegg M.A., Spier T.W. (2006). Longitudinal patterns of the Illinois waterway fish community. J. Fresh. Ecol..

[84] Beecher H.A., Dott E.R., Fernau R.F. (1988). Fish species richness and stream order in Washington State streams. Environ. Biol. Fish..

[85] Mandrak N.E. (1995). Biogeographic patterns of fish species richness in Ontario lakes in relation to historical and environmental factors. Can. J. Fish. Aquat. Sci..

[86] Oberdorff T., Guégan J.F., Hugueny B. (1995). Global scale patterns of fish species richness in rivers. Ecography.

[87] Joy M.K., Death R.G. (2002). Predictive modelling of freshwater fish as a biomonitoring tool in New Zealand. Freshwater Biol..

[88] Allan J.D., Castillo M.M. (2007). Reference. Stream Ecology: Structure and Function of Running Waters.

[89] Rosenberg D.M., Resh V.H. (1993). Reference. Freshwater Biomonitoring and Benthic Macroinvertebrates.

[90] Wiley M., Kohler S., Seelbach P. (1997). Reconciling landscape and local views of aquatic communities: Lessons from Michigan trout streams. Freshwater Biol..

[91] Johnson R.K., Furse M.T., Hering D., Sandin L. (2007). Ecological relationships between stream communities and spatial scale: Implications for designing catchmentlevel monitoring programmes. Freshwater Biol..

[92] Mori T., Murakami M., Saitoh T. (2010). Latitudinal gradients in stream invertebrate assemblages at a regional scale on Hokkaido Island, Japan. Freshwater Biol..

[93] Jowett I.G., Richardson J.S. (1990). Microhabitat preferences of benthic invertebrates in a New Zealand river and the development of in-stream flow-habitat models for *Deleatidium*. spp. New Zeal. J. Mar. Fresh..

[94] Eitzmann J.L., Paukert C.P. (2010). Longitudinal differences in habitat complexity and fish assemblage structure of a great plains river. Am. Midl. Nat..

[95] Townsend C.R., Hildrew A.G., Francis J. (1983). Community structure in some English streams: The influence of physicochemical factors. Freshwater Biol..

